# Health of Louisville

**Published:** 1853-06

**Authors:** 


### HEALTH OF LOUISVILLE.

Up to this time, (22d of June) the city remains almost entirely exempt from the complaints of hot weather, although the temperature has been remarkably high for the season. Several days this month the termometer in the shade has risen to 95 degrees, which is a good deal higher than ever we saw it before in June. An exceedingly severe drought is now prevailing. For more than five weeks scarcely any rain has fallen. Some cases of erysipelas have occurred in the city. Cholera excites no apprehensions, and is not heard of in any of the States. We hope it is gone from our country. It has afflicted us as long since its second visitation as it did when epidemic here before, and having, in so many instances, conformed to the course which it then pursued, we may hope that in this particular also it will not depart from it.

